# The relationship between spinal and pelvic parameters in the Japanese patients with adolescent idiopathic scoliosis

**DOI:** 10.1186/1748-7161-10-S1-O42

**Published:** 2015-01-19

**Authors:** Kenyu Ito, Shiro Imagama, Zenya Ito, Kei Ando, Kazuyoshi Kobayashi, Junichi Ukai, Akio Muramoto, Naoki Ishiguro

**Affiliations:** 1 Department of Orthopaedic Surgery, Nagoya University Hospital, Graduate School of Medicine, Japan

## Introduction

Several studies have shown that sagittal pelvic morphology influences the sagittal spinal morphology in normal adults. However, the relationship between pelvic and spinal parameters throughout the growth process in children is poorly defined in Japan. This study aimed to clarify the influence of pelvic parameters on spinal parameters in the Japanese children with idiopathic scoliosis.

## Methods

A total of 48 consecutive patients with idiopathic scoliosis (all female, mean age 15.9 years, range 12-20 years) were recruited from a single institution. All patients underwent standing posteroanterior and lateral radiographs of the spine and pelvis with their fists overlying the ipsilateral clavicle. Pelvic incidence (PI), sacral slope (SS), and pelvic tilt (PT) were measured as the pelvic parameters. Thoracic kyphosis (TK) and lumbar lordosis (LL) were measured as the sagittal spinal parameters. We divided the patients with scoliosis into hypokyphosis thoracic (H) and normal kyphosis (N) groups.

## Result

The mean angles were the Cobb angle 54.7 degrees, TK 14.0 degrees, LL 49.7 degrees, PI 46.9 degrees, SS 35.8 degrees, and PT 11.1 degrees, respectively. There was no correlation between the coronal Cobb angle and the pelvic parameters, whereas PI and TK (R=0.5), TK and LL (R=0.63), and PI and LL (R=0.7) were significantly correlated. The PI in the H group (41.4 degrees) was significantly smaller than in the N group (50.9 degrees).

## Conclusion

PI, LL, TK were correlated. Whereas, Cobb angle had no influence on pelvic parameter. These results suggested that patients with a lower PI may not grow into normal thoracic kyphosis or patients with hypokyphosis may not grow into a normal PI in the Japanese scoliosis patients.

